# Comparison of Phakic Intraocular Lens Vault Using Conventional Nomogram and Prediction Formulas

**DOI:** 10.3390/jcm9124090

**Published:** 2020-12-18

**Authors:** Wakako Ando, Kazutaka Kamiya, Hideki Hayakawa, Masahide Takahashi, Nobuyuki Shoji

**Affiliations:** 1 Department of Ophthalmology, School of Medicine, Kitasato University, Kanagawa 252-0374, Japan; wakako@kitasato-u.ac.jp (W.A.); m09088w@st.kitasato-u.ac.jp (H.H.); zavide96@gmail.com (M.T.); nshoji@kitasato-u.ac.jp (N.S.); 2 Visual Physiology, School of Allied Health Sciences, Kitasato University, Kanagawa 252-0373, Japan

**Keywords:** vault, ICL, phakic IOL, sizing, predictability, nomogram

## Abstract

This study aimed to compare the achieved vault using a manufacturer’s nomogram and the predicted vault using the currently available prediction formulas after posterior chamber phakic intraocular lens (EVO Implantable Collamer Lens; ICL, STAAR Surgical) implantation. We included 200 eyes of 100 consecutive patients (mean age ± standard deviation, 34.3 ± 7.8 years) undergoing ICL implantation with a central hole. Three months postoperatively, we quantitatively measured the actual vault, and we compared it with the predicted vault using anterior segment optical coherence tomography (CASIA 2, Tomey). The agreement rate of the recommended ICL size using the manufacturer’s nomogram, the NK formula, and the KS formula was 50.0%. The achieved vault was 477.1 ± 263.7 µm, which was significantly smaller than the predicted vaults of 551.2 ± 335.1 and 606.4 ± 212.2 µm, using the NK and KS formulas, respectively (Dunnett test, *p* = 0.014, *p* < 0.001). The achieved vault was not significantly different from the predicted vault using the NK or KS formula (*p* = 0.386, *p* = 0.157) when selecting a 12.1 mm ICL size. It was not significantly different from the predicted vault using the NK formula (*p* = 0.962), but it was significantly smaller than that using the KS formula (*p* = 0.033) when selecting a 12.6 mm size. It was significantly smaller than the predicted vault using the NK and KS formulas (*p* < 0.001) when selecting 13.2 mm size. The total agreement rate of the recommended ICL size was approximately 50%. The predicted ICL vault tended to overestimate the actual ICL vault, especially when selecting a larger ICL size.

## 1. Introduction

The EVO Implantable Collamer Lens (ICL; Visian ICL with KS-AquaPORT; STAAR Surgical, Monrovia, CA, USA), a posterior chamber phakic intraocular lens, has been well-recognized as a long-term, safe, and effective means to correct moderate to high refractive errors all over the world [1,2,3,4]. Nevertheless, angle closure and subsequent intraocular pressure elevation can occur when selecting a larger ICL size, whereas cataract formation or toric ICL rotation can occur when selecting a smaller ICL size. Accordingly, there are still ongoing concerns about selecting the optimal ICL size, even when applying the recommended ICL sizing nomogram provided by the manufacturer, based on measurements of the white-to-white diameter (WTW) and the anterior chamber depth (ACD).

Since the ICL is fixated into the ciliary sulcus, direct sulcus-to-sulcus (STS) measurements are theoretically ideal for selecting the proper ICL size. Although ultrasound biomicroscopy (UBM) can directly measure the STS, this measurement is time-consuming and subject to the examiner’s skills and experience; thus, it is not commonly used in daily practice. In recent years, swept source anterior segment optical coherence tomography (AS-OCT) has been shown to provide more precise measurements of the anterior segment biometrics, especially the angle-to-angle distance (ATA), than other anterior segment biometric devices, such as UBM [5,6]. Currently, the NK and KS formulas have become commercially available for predicting the ICL vault and selecting the optimal ICL size, based on an AS-OCT device (CASIA2, Tomey, Nagoya, Japan) [7,8,9]. However, to the best of our knowledge, the achieved and predicted ICL vault using these formulas has, to date, not been validated in any other population. It may give us essential prospects on the practical use of the two prediction formulas in a clinical setting. The purpose of the current study was twofold: to determine the agreement rate of the recommended ICL size using the manufacturer’s nomogram and the two prediction formulas; and to compare the achieved and predicted ICL vault using the two formulas according to the ICL size actually selected.

## 2. Patients and Methods

### 2.1. Study Population

We registered the study protocol with the University Hospital Medical Information Network Clinical Trial Registry (000041287). This study comprised 200 eyes of 100 consecutive patients (47 men, 53 women) who underwent EVO ICL implantation (V4c and V5 Visian ICL with KS-AquaPORT, V4c; 100 eyes, V5; 100 eyes) (non-toric model, 116 eyes; toric model, 84 eyes) for the correction of moderate to high myopia and myopic astigmatism, and who completed at least a 3-month follow-up at Kitasato University Hospital, in this case series. The inclusion criteria for ICL surgery at our institution include unsatisfactory correction with spectacles or contact lenses, 20 ≤ age ≤ 50 years, stable refraction for at least 3 months, −3.00 to −14.0 diopters (D) of myopia with astigmatism of 3 D or less, anterior chamber depth ≥ 2.8 mm, endothelial cell density ≥ 1800 cells/mm^2^, and no history of ocular surgery, corneal diseases, cataract, glaucoma, or uveitis. Keratoconic eyes were excluded from this study on the basis of a screening test (TMS-5^TM^, Tomey, Nagoya, Japan). The sample size in the present study yielded 94.1% statistical power at the 5% level to detect a 75 µm difference in the ICL vault, and the standard deviation (SD) of the mean difference was 300 µm. This retrospective clinical chart review was approved by the Institutional Review Board at Kitasato University Hospital (B20-111), and it adhered to the principles of the Declaration of Helsinki. Written informed consent was obtained from all patients for this surgery.

### 2.2. Lens Size Selection and Power Calculation

We selected the appropriate ICL size (12.1, 12.6, 13.2, or 13.7 mm) using the manufacturer’s nomogram based on the WTW and ACD measurements. We determined the ICL power using an online calculator system, provided by the manufacturer, based on a modified vertex formula.

### 2.3. Surgical Procedure

Detailed surgical procedures were described previously in our studies [10,11]. In brief, on the day of surgery, dilating and topical anesthetic agents were applied. A model V4c or V5 ICL was implanted through a 3 mm temporal clear corneal incision after injecting a viscoelastic substance into the anterior chamber. The ICL was inserted into the posterior chamber, the viscoelastic substance was replaced with a balanced salt solution, and a miotic agent was administrated. Both non-toric and toric ICLs were implanted horizontally in accordance with the manufacturer’s recommendations. None of the toric ICLs required a rotation of 10 degrees or more from the horizontal meridian in this series. We used topical steroidal (0.1% betamethasone) and antibiotic (1.5% levofloxacin) medications 4 times daily for 1 week, with the dose being reduced gradually thereafter.

### 2.4. Predicted and Achieved Vault Assessment

Preoperatively, we obtained the anterior segment biometric data using an AS-OCT (CASIA2^TM^, Tomey, Nagoya, Japan) under bright light conditions (500 lux). The device was equipped with the currently available prediction formulas (the NK formula version 2 [8] and the KS formula [9]) and automatically calculated the predicted ICL vault by the use of these biometric data, such as ATA, anterior chamber width (ACW), and crystalline lens rise (CLR). Three months postoperatively, we automatically measured the amount of the central ICL vault, using the same AS-OCT under the same conditions. Patients were asked to blink immediately before starting the AS-OCT measurements. After that, we first checked the image quality and selected one examination with high image quality.

### 2.5. Repeatability Assessment

Additionally, to validate the repeatability of the AS-OCT measurements, the ICL vault measurements were performed at the same time of the day on two consecutive days in 20 ICL-implanted eyes. The repeatability of the two measurements was evaluated using Bland–Altman plots, as described previously [12].

### 2.6. Statistical Analysis

One-way analysis of variance (ANOVA) was used to analyze the three groups, with the Dunnett test being employed for multiple comparisons. The chi-squared test was used to compare the agreement rate of the recommended ICL. Unless otherwise indicated, the results are expressed as mean ± SD, and a value of *p* < 0.05 was considered to be statistically significant.

## 3. Results

Table 1 summarizes preoperative and postoperative demographic information of the study population. All surgeries were uneventful, and no vision-threatening complications such as cataracts, pupillary block, intraocular pressure rise, or uveitis occurred at any time. No eyes required ICL exchange due to extremely low or high ICL vault in this series.

The total agreement rate of the recommended ICL size using the manufacturer’s nomogram, the NK formula, and the KS formula was 50.0%. The disagreement rates of the recommended ICL size using the manufacturer’s nomogram and the NK formula, and using the nomogram and the KS formula, were 42.5% and 37.5%, respectively (chi-squared test, *p* = 0.519). The NK and KS formulas selected a smaller ICL size than the nomogram in 53 eyes (26.5%) and 66 eyes (33.0%), respectively (chi-squared test, *p* < 0.001). The agreement rate of the recommended ICL size using the NK and KS formulas was 76.5% (15 eyes for a 12.1 mm, 70 eyes for a 12.6 mm, and 68 eyes for a 13.2 mm ICL size). The recommended ICL size using the NK formula was larger and smaller than that using the KS formula in 44 eyes (22.0%) and 3 eyes (1.5%), respectively. Table 2 summarizes the achieved ICL vault using the manufacturer’s nomogram and the predicted ICL vault by using the NK and KS formulas, according to the selected ICL size. The number of selected ICL sizes (12.1, 12.6, 13.2, and 13.7 mm) according to the manufacturer’s nomogram were 21 eyes (10.5%), 95 eyes (47.5%), 83 eyes (41.5%), and 1 eye (0.5%), respectively. The achieved ICL vault was 477.1 ± 263.7 µm, which was significantly smaller than the predicted ICL vault of 551.2 ± 335.1 µm using the NK formula (Dunnett test, *p* = 0.014) and 606.4 ± 212.2 µm using the KS formula (*p* < 0.001) in the entire study population (ANOVA, *p* < 0.001).

According to the ICL size actually selected, when selecting a 12.1 mm ICL size, the achieved ICL vault of 257.9 ± 194.3 µm was not significantly different from the predicted ICL vault of 185.6 ± 265.2 µm using the NK formula (*p* = 0.386) or 175.0 ± 83.0 µm using the KS formula (*p* = 0.157). When selecting a 12.6 mm ICL size, the achieved ICL vault of 460.6 ± 245.1 µm was not significantly different from the predicted ICL vault of 467.7 ± 247.5 µm using the NK formula (*p* = 0.962), but it was significantly smaller than that of 535.0 ± 129.6 µm using the KS formula (*p* = 0.033). When selecting a 13.2 mm ICL size, the achieved ICL vault of 544.1 ± 262.8 µm was significantly smaller than the predicted ICL vault of 732.2 ± 325.3 µm using the NK formula (*p* < 0.001) and 743.1 ± 220.6 µm using the KS formula (*p* < 0.001).

Table 3 summarizes the achieved vault and the predicted vault by the NK and KS formulas for each ICL model (V4c and V5). The differences between the achieved vault and the predicted vault by the NK formula in V5-implanted eyes tended to be smaller than those in V4c-implanted eyes, but the differences between the achieved vault and the predicted vault by the KS formula tended to remain constant between V4c- and V5-implanted eyes.

**Figure 1:**
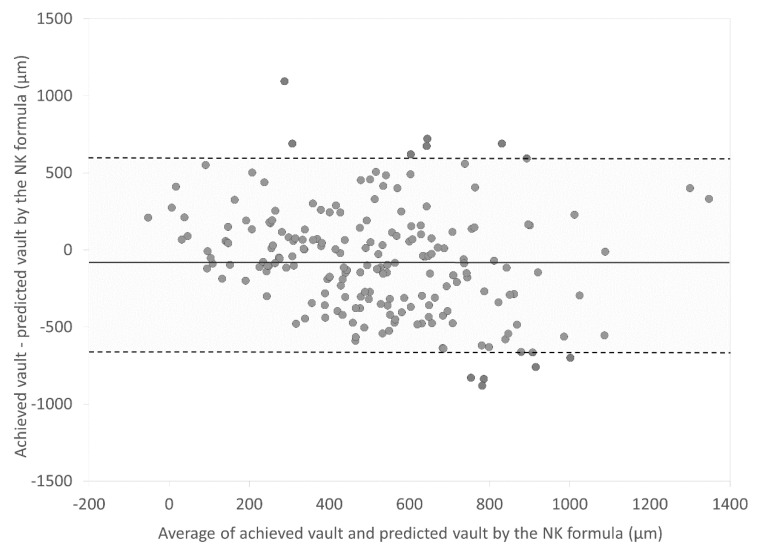
Bland–Altman plots showing the difference between the achieved vault and the predicted vault by the NK formula divided by the mean of the achieved vault and the predicted vault. The solid line represents the mean difference between two consecutive measurements of corneal astigmatism, dotted lines are the upper and lower borders of the 95% LoA (mean difference ± 1.96 multiplied by standard deviation of the mean difference).

**Figure 2:**
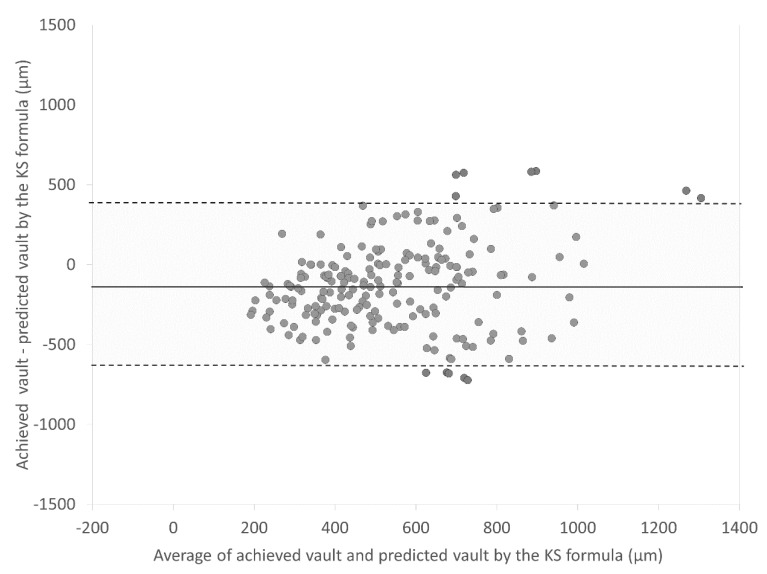
Bland–Altman plots showing the difference between the achieved vault and the predicted vault by the KS formula divided by the mean of the achieved vault and the predicted vault. The solid line represents the mean difference between two consecutive measurements of corneal astigmatism, dotted lines are the upper and lower borders of the 95% LoA (mean difference ± 1.96 multiplied by standard deviation of the mean difference).

Figure 1 and Figure 2 are Bland–Altman plots of the achieved vault and the predicted vault using the NK and KS formulas, respectively. Mean differences between the achieved vault and the predicted vault using the NK and KS formulas were −74.2 ± 338.7 µm and −129.4 ± 264.6 µm, respectively (*p* < 0.001). The 95% limits of agreement of the achieved vault and the predicted vault using the KS formula were narrower than those of the achieved vault and the predicted vault using the NK formula.

## 4. Discussion

In the current study, our findings showed that the agreement rates of the recommended ICL size using the manufacturer’s nomogram and the NK formula, and the rate using the nomogram and the KS formula, were 57.5% and 62.5%, respectively, and the total agreement rate of the recommended ICL size among the three methods was only 50.0% in the entire study population. It should be noted that both prediction formulas tend to select a smaller ICL size than the actual nomogram in a clinical setting. Our findings also demonstrated that the achieved postoperative ICL vault was significantly smaller than the predicted ICL vault using the NK and KS formulas in the study population, and the predicted ICL vault tended to overestimate the actual ICL vault, especially when selecting a larger ICL size (13.2 mm size). To the best of our knowledge, this is the first study to externally validate the achieved and predicted ICL vault using the NK and KS formulas, and the recommended ICL size using the manufacturer’s nomogram and the two prediction formulas in current ICL-implanted eyes. It should be noted that the predicted ICL vault was almost the same as the actual ICL vault, but the two prediction formulas still need to be modified, especially when a larger ICL size is selected.

The manufacturer’s nomogram recommends the optimal ICL size, based only on WTW and ACD measurements, to make the targeted ICL vault identical to corneal thickness (approximately 500 µm). By contrast, both the NK and KS formulas are based on the anterior segment metrics obtained by the AS-OCT to predict the ICL vault, according to each ICL size, and to recommend the optimal ICL size.

According to the NK formula [7,8], the optimal ICL size and the predicted ICL vault were calculated using the following equation: optimal ICL size = 4.575 + 0.688 × ACW + 0.388 × CLR, where ACW is defined as the distance between the scleral spurs on the nasal and temporal sides, CLR is defined as the anteroposterior distance between the anterior crystalline lens surface and the angle recess-to-angle recess line, and ICL vault = 0.5 + 1.1 × (implanted ICL size − optimal ICL size using the NK formula). According to the KS formula [9], the predicted ICL vault was calculated using the following equation: predicted ICL vault (µm) = 660.9 × (ICL size (mm) − ATA (mm)) + 86.6. However, both prediction formulas were calculated on the basis of the AS-OCT biometric data with a small sample size and included all ICL sizes (12.1, 12.6, 13.2, and 13.7 mm) for creating the equation. We found that the NK formula tended to recommend a larger ICL size than the KS formula when the recommended ICL size of the two prediction formulas was inconsistent. On the basis of our findings that the actual ICL vault was substantially influenced by the ICL size itself, we assume that these existing prediction formulas will need to be modified according to each ICL size to further improve the prediction accuracy in a clinical setting.

The NK and KS prediction formulas have been optimized for V4c-implanted eyes and V4c- and V5-implanted eyes, respectively. Nevertheless, on the basis of our results of the ICL models, the NK formula in V5-implanted eyes provided slightly better outcomes than that in V4c-implanted eyes, whereas the KS formula provided similar outcomes in V4c- and V5-implanted eyes. Considering that the V4c and V5 models have design differences that may cause varying mechanical properties and positioning in the eye, further investigation is necessary to clarify this point. No eyes developed cataracts, pupillary block, or intraocular pressure rise, nor did any eyes require ICL exchange due to an extremely low or high ICL vault in this study population. In principle, we carefully monitor the intraocular pressure rise and the corneal endothelial cell loss in eyes having an extremely high ICL vault. We also monitor the mechanical contact between the ICL and the crystalline lens and subsequent cataract formation in eyes having an extremely low ICL vault. We consider ICL exchange (one size down or up) only when we find symptoms for these abnormalities.

There were at least the two limitations to our study. First, this study was performed in a retrospective fashion. A prospective study may provide further information for confirming the authenticity of these results. Second, we included both eyes of the same patient in this study, although only one eye should be used for statistical analysis in consideration of the inter-eye correlations [13]. We confirmed similar results, even when only one eye was randomly chosen from each patient, suggesting that the bilateral issue might not be particularly severe (as the problem of including both eyes is mostly exaggerating type 1 errors) in this study population. Thus, we enrolled both eyes of the same patient in this case series as described in many published studies on refractive surgery.

## 5. Conclusions

In summary, our study showed that the total agreement rate of the recommended ICL size among the three methods was only 50.0%. The achieved ICL vault was significantly smaller than the predicted ICL vault using the NK and KS formulas, and the differences tended to be larger when a larger ICL size was selected. Our results may support the view that the NK and KS prediction formulas should be optimized for individual ICL sizes. A further modification of these prediction formulas according to each selected ICL size is still necessary to improve the predicted ICL vault accuracy in ICL-implanted eyes.

## Figures and Tables

**Table 1:** Preoperative and postoperative demographics of the study population undergoing Implantable Collamer Lens (ICL) implantation.

Demographic	Preoperative	Postoperative (3 Months)	*p*-Value
Age (years)	34.3 ± 7.8 (95%CI, 19.2 to 49.4)		
Gender (male:female)	47:53		
Manifest spherical refraction (D)	−6.98 ± 3.46 (95%CI, −13.76 to −0.21)	−0.10 ± 0.44 (95%CI, −0.96 to 0.77)	<0.001
Manifest cylinder (D)	−1.08 ± 1.09 (95%CI, −3.22 to 1.66)	−0.24 ± 0.37 (95%CI, −0.97 to 0.49)	0.004
UDVA (logMAR)	1.28 ± 0.29 (95%CI, 0.71 to 1.86)	−0.11 ± 0.19 (95%CI, −0.24 to 0.46)	<0.001
CDVA (logMAR)	−0.16 ± 0.12 (95%CI, −0.40 to 0.08)	−0.22 ± 0.46 (95%CI, −0.05 to −0.38)	0.108
Intraocular pressure (mmHg)	14.2 ± 2.6 (95%CI, 9.1 to 19.3)	14.0 ± 2.5 (95%CI, 9.0 to 18.9)	0.087
Endothelial cell density (cells/mm^2^)	2823 ± 241 (95%CI, 2350 to 3295)	2790 ± 220 (95%CI, 2358 to 3222)	0.158

D = diopter, CI = confident interval, logMAR = logarithm of the minimal angle of resolution, UDVA = uncorrected distance visual acuity, CDVA = corrected distance visual acuity.

**Table 2:** The achieved ICL vault using the manufacturer’s nomogram and the predicted ICL vault by the NK and KS formulas according to the selected ICL size.

ICL Size	Eyes (%)	Achieved ICL Vault	Predicted ICL Vault Using the NK Formula	Predicted ICL Vault Using the KS Formula
Total	200 (100%)	477.1 ± 263.7 µm	551.2 ± 335.1 µm (*p* = 0.014)	606.4 ± 212.2 µm (*p* < 0.001)
12.1 mm	21 (10.5%)	257.9 ± 194.3 µm	185.6 ± 265.2 µm (*p* = 0.386)	175.0 ± 83.0 µm (*p* = 0.157)
12.6 mm	95 (47.5%)	460.6 ± 245.1 µm	467.7 ± 247.5 µm (*p* = 0.962)	535.0 ± 129.6 µm (*p* = 0.033)
13.2 mm	83 (41.5%)	544.1 ± 262.8 µm	732.2 ± 325.3 µm (*p* < 0.001)	743.1 ± 220.6 µm (*p* < 0.001)
13.7 mm	1 (0.5%)	1083 µm	1093 µm	909 µm

ICL = Implantable Collamer Lens, Dunnett test.

**Table 3:** The achieved vault using the manufacturer’s nomogram and the predicted vault by NK and KS formulas for each model (V4c and V5).

ICL Size	Eyes (%)	Achieved ICL Vault	Predicted ICL Vault Using the NK Formula	Predicted ICL Vault Using the KS Formula
**V4c model (100 eyes)**				
Total	100 (100%)	496.1 ± 334.4 µm	662.8 ± 366.2 µm (*p* < 0.001)	673.4 ± 232.9 µm (*p* < 0.001)
12.1 mm	13 (13.0%)	163.4 ± 119.6 µm	197.1 ± 248.8 µm (*p* = 0.817)	380.5 ± 47.3 µm (*p* = 0.003)
12.6 mm	45 (45.0%)	386.3 ± 198.7 µm	450.6 ± 283.0 µm (*p* = 0.161)	529.1 ± 142.9 µm (*p* = 0.004)
13.2 mm	42 (42.0%)	539.5 ± 283.4 µm	846.0 ± 309.2 µm (*p* < 0.001)	806.4 ± 226.6 µm (*p* < 0.001)
13.7 mm	0 (0%)	-	-	-
**V5 model (100 eyes)**				
Total	100 (100%)	489.5 ± 236.3 µm	583.9 ± 327.0 µm (*p* = 0.894)	635.2 ± 212.2 µm (*p* = 0.211)
12.1 mm	8 (8.0%)	411.4 ± 199.5 µm	166.9 ± 306.9 µm (*p* = 0.072)	366.0 ± 125.3 µm (*p* = 0.820)
12.6 mm	50 (50.0%)	527.5 ± 264.9 µm	483.2 ± 212.3 µm (*p* = 0.460)	540.3 ± 117.5 µm (*p* = 0.932)
13.2 mm	41 (41.0%)	548.7 ± 243.4 µm	616.8 ± 302.9 µm (*p* = 0.223)	678.3 ± 196.6 µm (*p* = 0.040)
13.7 mm	1 (1.0%)	1083 µm	1093 µm	909 µm

## Abbreviations

ICL	Implantable Collamer Lens
WTW	white-to-white diameter
ACD	anterior chamber depth
STS	sulcus-to-sulcus diameter
UBM	ultrasound biomicroscopy
AS-OCT	anterior segment optical coherence tomography
ATA	angle-to-angle distance
D	diopter
SD	standard deviation
ACW	anterior chamber width
CLR	crystalline lens rise
ANOVA	analysis of variance
LoA	limits of agreement
